# ECG Markers of Hemodynamic Improvement in Patients with Pulmonary Hypertension

**DOI:** 10.1155/2018/4606053

**Published:** 2018-04-10

**Authors:** Marcin Waligóra, Anna Tyrka, Piotr Podolec, Grzegorz Kopeć

**Affiliations:** Department of Cardiac and Vascular Diseases, Jagiellonian University Medical College, John Paul II Hospital in Krakow, Pradnicka 80, Kraków, Poland

## Abstract

**Introduction:**

Several diagnostic tests have been recommended for risk assessment in pulmonary hypertension (PH), but the role of electrocardiography (ECG) in monitoring of PH patients has not been yet established. Therefore the aim of the study was to evaluate which ECG patterns characteristic for pulmonary hypertension can predict hemodynamic improvement in patients treated with targeted therapies.

**Methods:**

Consecutive patients with pulmonary arterial hypertension (PAH) or chronic thromboembolic pulmonary hypertension (CTEPH) were eligible to be included if they had had performed two consecutive right heart catheterization (RHC) procedures before and after starting of targeted therapies. Patients were followed up from June 2009 to July 2017. ECG patterns of right ventricular hypertrophy according to American College of Cardiology Foundation were assessed.

**Results:**

We enrolled 80 patients with PAH and 11 patients with inoperable CTEPH. The follow-up RHC was performed within 12.6 ± 10.0 months after starting therapy. Based on median change of pulmonary vascular resistance, we divided our patients into two subgroups: with and without significant hemodynamic improvement. *R*_*V*1_, max⁡*R*_*V*1,2_ + max⁡*S*_I,aVL_ − *S*_*V*1_, and *P*_II_ improved along with the improvement of hemodynamic parameters including PVR. They predicted hemodynamic improvement with similarly good accuracy as shown in ROC analysis: *R*_*V*1_ (AUC: 0.75; 95% CI: 0.63–0.84), *P*_II_ (AUC: 0.67, 95% CI: 0.56–0.77), and max⁡*R*_*V*1,2_ + max⁡*S*_I,aVL_ − *S*_*V*1_ (0.73; 95% CI: 0.63–0.82). In Cox regression only change in *R*_*V*1_ remained significant mortality predictor (HR: 1.12, 95% CI: 1.01–1.24).

**Conclusion:**

Electrocardiogram may be useful in predicting hemodynamic effects of targeted therapy in precapillary pulmonary hypertension. Decrease of *R*_*V*1_, max⁡*R*_*V*1,2_ + max⁡*S*_I,aVL_ − *S*_*V*1_, and *P*_II_ corresponds with hemodynamic improvement after treatment. Of these changes a decrease of *R* wave amplitude in *V*_1_ is associated with better survival.

## 1. Introduction

Pulmonary hypertension (PH) is a severe progressive disease characterized by narrowing and occlusion of pulmonary arteries. The current treatment algorithm requires frequent assessment of the patient and escalation of therapy if low risk status has not been achieved. Several diagnostic tests have been recommended for risk evaluation including physical examination, assessment of World Heart Organization functional class (WHO-FC), 6-minute walk test (6 MWT), cardiopulmonary exercise testing, N-terminal pro-B type natriuretic peptide level (NT-proBNP), imaging studies, and right heart catheterization (RHC) [1]. Till now the role of electrocardiography (ECG) in monitoring of PH patients has not been established.

Chronic overload of the right ventricle (RV) as observed in PH leads to RV hypertrophy and dilation. This can be reflected by several patterns in surface electrocardiogram. Hemodynamic, autopsy, and cardiac magnetic resonance imaging (cMR) studies have shown significant correlations between hemodynamic burden, right ventricular hypertrophy, and ECG changes as reported in several studies [2–9]. Little is known however about whether hemodynamic improvement observed in treated PH patients can be reflected by ECG changes.

Therefore the aim of the study was to evaluate which ECG patterns characteristic for pulmonary hypertension can predict hemodynamic improvement in patients treated with targeted therapies.

## 2. Methods

### 2.1. Patients

All study participants were selected from a cohort of patients with PH diagnosed and treated in our centre between June 2009 and March 2016. Patients were eligible if they had pulmonary arterial hypertension (PAH) or inoperable chronic thromboembolic pulmonary hypertension (CTEPH) with RHC and ECG performed on the same day at least twice: the first before starting or escalating targeted therapy and the second at least 3 months later. Pulmonary hypertension was defined as a mean pulmonary artery pressure (mPAP) ≥ 25 mmHg at rest as assessed by right heart catheterization. PAH was defined as precapillary pulmonary hypertension (pulmonary artery wedge pressure ≤ 15 mmHg) with pulmonary vascular resistance > 3 Wood units in the absence of other causes of precapillary PH such as lung diseases, chronic thromboembolic pulmonary hypertension, or other rare diseases [1]. CTEPH was defined as precapillary pulmonary hypertension (pulmonary artery wedge pressure ≤ 15 mmHg) after at least 3 months of effective anticoagulation with a presence of mismatched perfusion defects on lung scan or multidetector CT angiography [1]. The operability assessment in all CTEPH patients was performed by a multidisciplinary CTEPH team. Both treatment-naive patients (patients who have not been previously treated with therapies specific for pulmonary arterial hypertension) and patients already treated with PAH targeted medications could have been included. Main exclusion criteria were age < 18 years and lack of informed consent. Clinical assessment included demographic information, patient's medical history, NT-proBNP, 6 MWT distance (6 MWD), assessment of the WHO-FC, resting 12-lead ECG, and RHC. Patients were treated with PAH specific drugs according to European Society of Cardiology (ESC) guidelines [1] and local standards. All-cause mortality was ascertained by data collection (1) from medical registry of hospital, (2) from the Department of Nationals' and Foreigners' Affairs, or (3) through phone follow-up. Patients were enrolled between June 2009 and March 2016 and the observation period was extended until July 2017. The baseline assessment was at the time of RHC which resulted in diagnosis of PH or escalation of targeted therapy. The study protocol conforms to the ethical guidelines of the 1975 Declaration of Helsinki and was approved by the institutional ethics committee. Informed consent was obtained from each patient before starting the study.

### 2.2. Electrocardiography

A 12-lead standard ECG (10 mm = 1 mV, 25 mm/s) was acquired in a supine position during quiet respiration. For the purpose of the present study we assessed several parameters proposed by American College of Cardiology Foundation and the Heart Rhythm Society (AHA/ACCF/HRS) [10] to diagnose RV hypertrophy. We assessed quantitative parameters: *R*_*V*1_, *R* : *S*_*V*1_, *S*_*V*5_, *S*_*V*6_, *R*_aVR_, *S*_*V*1_, *R*_*V*5,6_, *R* : *S*_*V*5_, *R* : *S*_*V*6_, *R* : *S*_*V*5_ to *R* : *S*_*V*1_, (*R*_I_ + *S*_III_) − (*S*_I_ + *R*_III_), max⁡*R*_*V*1,2_ + max⁡*S*_I,aVL_ − *S*_*V*1_, *R*_*V*1_ + *S*_*V*5,6_, *R* peak *V*_1_ (QRS duration < 0.12 sec), and *P*_II_, as well as qualitative patterns: presence of QR in *V*_1_, RSR_*V*1_ (QRS duration > 0.12 sec), *S* > *R*_I,II,III_, *S*_I_ and *Q*_III_, *R* : *S*_*V*1_ > *R* : *S*_*V*3,4_, and negative *T*-waves in leads *V*_1_–*V*_3_. Right bundle branch block (RBBB) was as recommended [11]: QRS duration ≥ 120 ms, with typical QRS morphology in *V*_1_ or *V*_2_ (rsr', rsR', rSR', wide and notched *R*), and *S* wave duration > *R* wave duration or >40 ms in I and *V*_6_. When a pure dominant *R* wave with or without a notch was present in *V*_1_, addition criterion had to be satisfied: normal *R* peak time in *V*_5_ and *V*_6_ but >50 ms in *V*_1_.

### 2.3. Right Heart Catheterization

RHC was performed in a supine position from the right femoral vein or right internal jugular vein access using a Swan-Ganz catheter. All measurements including acquisition of pressure waves were made at end expiration. Cardiac output was measured using the Fick direct oxygen consumption method. Blood oxygen saturation was measured with CO-oximeter OSM3 (Radiometer, Copenhagen, Denmark). Cardiac index (CI) was calculated as cardiac output divided by body surface area (BSA). BSA was calculated from the Mosteller formula [12]. Pulmonary vascular resistance (PVR) was calculated as the difference between mean pulmonary arterial pressure (mPAP) and pulmonary artery wedge pressure divided by cardiac output. Based on a median change of PVR in all study patients we distinguished two subgroups: with and without hemodynamic improvement.

### 2.4. Statistics

Continuous variables are reported using means and standard deviations. Categorical variables are described as counts and percentages. Continuous variables were compared using the Student *t*-test or Mann–Whitney *U* test when appropriate. The *χ*^2^-test was used to compare categorical variables. McNemar's test was used to compare paired data of meeting right ventricular hypertrophy criteria before and after addition of targeted therapy. The Bonferroni correction was applied when changes in several ECG patterns were compared between patients with and without hemodynamic improvement. The relationship between changes of ECG and hemodynamics was estimated by Pearson or Spearman correlation tests. Several receiver-operating characteristics (ROC) curves were drawn to compare the accuracy of changes in different ECG patterns in predicting hemodynamic improvement. Univariate Cox regression analysis was used to assess significant associations between changes in ECG and survival. Statistical analysis was performed with Statistica PL software [Dell Inc. (2016), Dell Statistica (data analysis software system), version 13; software.dell.com] and MedCalc Statistical software version 16.8 (MedCalc software bvba, Ostend, Belgium; https://www.medcalc.org; 2016). The significance level was set at alpha level of 0.05.

## 3. Results

### 3.1. Patients

Between June 2009 and March 2016, 158 patients were diagnosed with PAH and 46 with CTEPH. Among them 80 patients with PAH and 11 with inoperable CTEPH were included in the present analysis. Excluded PAH patients did not have follow-up RHC (Eisenmenger's syndrome; *n* = 55, lack of consent; *n* = 2, lost to follow-up; *n* = 1, premature death; *n* = 12), permanent pacemaker stimulation (*n* = 5), or insufficient quality of ECG recordings (*n* = 3). We excluded the CTEPH patients who were referred to pulmonary endarterectomy or pulmonary balloon angioplasty.

Overall the study sample included 91 patients aged 52.6 ± 16.4 (68.1% females). The patients had idiopathic PAH (IPAH; *n* = 54, 59.3%), PAH associated with connective tissue disease CTD-APAH (*n* = 16, 17.6%), and PAH associated with congenital heart diseases (CHD-APAH; *n* = 10, 11%) or inoperable CTEPH (*n* = 11, 12.1%). Patients were in WHO-FC II (*n* = 9, 9.9%), III (*n* = 61; 67%), or IV (*n* = 21, 23.1%) at initial assessment. Majority of patients were newly diagnosed and treatment-naive (*n* = 71, 78%) and were initially treated with a monotherapy of either phosphodiesterase-5 inhibitor (PDE-5i) in 35 (49.2%), endothelin receptor antagonist (ERA) in 14 (17.7%), parenteral prostacyclin analogue in 10 (14.1%), inhaled iloprost 4 (5.6%), or calcium channel blocker in 8 (11.3%). The other patients (*n* = 20) were already treated with targeted therapies: riociguat, 1 (1.1%); ERA, 4 (4.4%); PDE-5i, 9 (9.9%); PDE-5i and ERA, 4 (4.4%); PDE-5i and inhaled iloprost, 1 (1.1%); and inhaled iloprost and ERA, 1 (1.1%). In this group follow-ups were gathered after addition of the following treatment: parenteral prostacyclin analogue in 17 (85%), PDE-5i in 1 (5%), ERA in 1 (5%), and inhaled iloprost in 1 (5%).

The follow-up RHC was performed within 12.6 ± 10.0 months after study enrollment. The median change of pulmonary vascular resistance was −2.1 [−4.5; 0.7] Wood Units which was 17% of the baseline PVR value. Based on this value, we divided our patients into two groups: with significant hemodynamic improvement (decrease of PVR ≥ 17% from baseline value, *n* = 46) and without significant hemodynamic improvement (decrease of PVR < 17% from baseline value, *n* = 46). Baseline clinical and hemodynamic characteristics of these two subgroups were similar as shown in Table 1. Patients with hemodynamic improvement had added parenteral prostacyclin analogues more frequently than patients without hemodynamic improvement [18 (39.1%) versus 9 (20%), *p* = 0.05]. No differences were observed with reference to other therapies: PDE-5 inhibitors [16 (34.5%) versus 20 (44.4%), *p* = 0.35], ERA [5 (10.9%) versus 10 (22.2%), *p* = 0.15], inhaled iloprost [3 (6.5%) versus 2 (4.4%) versus, *p* = 0.67], or CCB [4 (8.7%) versus (8.9%), *p* = 0.97]. After addition of specific treatment we found the following changes of hemodynamic parameters in a group with and without hemodynamic improvement, respectively: mPAP −8.7 ± 10.8 mmHg (*p* < 0.001) and +0.8 ± 11.3 mmHg (*p* = 0.64), cardiac index +0.59 ± 0.9 l/kg/m2 (*p* < 0,001) and −0.14 ± 0.56 l/kg/m2 (*p* = 0.1), mRAP −2.5 ± 5.5 mmHg (*p* = 0.02) and −0.9 ± 4,2 mmHg (*p* = 0.45), and PVR −5.4 ± 4.5 WU (*p* < 0.01) and +1.9 ± 4.8 WU (*p* = 0.01).

### 3.2. ECG Changes after Treatment

**Figure 1:**
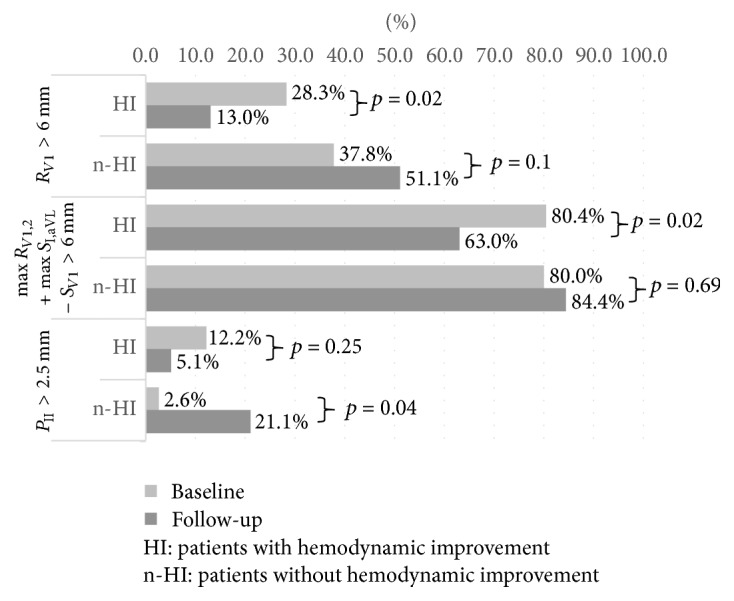
Changes of fulfilling right ventricular hypertrophy criteria in a study group before and after addition of targeted treatment.

**Figure 2:**
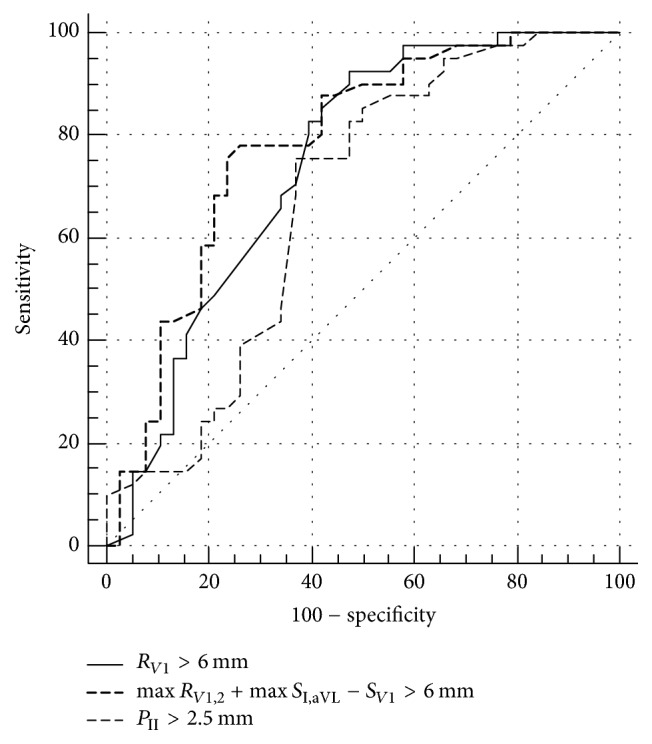
Electrocardiographic predictors of hemodynamic improvement. Δ*R*_*V*1_ (AUC: 0.75, 95% CI: 0.63–0.84, *p* = 0.0005), Δmax⁡*R*_*V*1,2_ + max⁡*S*_I,aVL_ − *S*_*V*1_ (AUC: 0.73, 95% CI: 0.63–0.82, *p* < 0.0001), and Δ*P*_II_ (AUC: 0.67, 95% CI: 0.56–0.77, *p* = 0.007).

Patients without complete or incomplete RBBB accounted for majority of the study group (*n* = 74, 81.3%). Twelve patients presented atrial fibrillation during initial assessment; therefore they did not have *P* wave amplitudes calculated (6 in group with significant hemodynamic improvement and 6 in group without it). Twenty-four patients had no S_*V*1_ and were not eligible for calculations of criteria using *S* wave. Baseline electrocardiographic characteristics of patients with and without hemodynamic improvement are showed in Table 2. The follow-up ECG showed that after addition of PAH specific therapy in the whole sample none of ECG parameters changed significantly. However when sample was divided into patients with and without hemodynamic improvement we found differences in changes of the following parameters: *R*_*V*1_, max⁡*R*_*V*1,2_+max⁡*S*_I,aVL_ − *S*_*V*1_, and *P*_II_ as shown in Table 3 and Table S1. Changes of fulfilling these RVH criteria before and after addition of targeted treatment are presented in Figure 1. Similar observations were also present when only patients without RBBB were assessed as presented in Tables S2 and S3. Changes in these ECG patterns correlated with changes of several hemodynamic parameters including ΔPVR, ΔmPAP, and ΔCI as shown in Table 4. In Figure 2. we compare three ROC analyses to show how changes in different ECG parameters predicted significant hemodynamic improvement in ROC analysis (*p* = 0.56 for comparison of Δ*R*_*V*1_ and Δmax⁡*R*_*V*1,2_+max⁡*S*_I,aVL_ − *S*_*V*1_, *p* = 0.4 for comparison of Δ*R*_*V*1_ and Δ*P*_II_, and *p* = 0.18 for comparison of Δmax⁡*R*_*V*1,2_ + max⁡*S*_I,aVL_ − *S*_*V*1_ and Δ*P*_II_).

The RBBB was diagnosed in 17 patients at baseline assessment (complete in 9 and incomplete in 8 patients). In this group we did not find any differences in changes of ECG patterns with and without hemodynamic improvement as presented in Tables S4 and S5.

### 3.3. Electrocardiography Changes and Long-Term Follow-Up

During prospective observation of a mean of 27.9 ± 9.5 months, 20 patients died (22.0%). In univariate Cox proportional hazard models, change in *R*_*V*1_ was significantly associated with mortality (HR: 1.12, 95% CI: 1.01–1.24, *p* = 0.02). Changes in the other criteria were not significantly associated with mortality. At follow-up *R*_*V*1_ increased in 36 (39.6%) patients, decreased in 48 (52.7%) patients, and remained unchanged in 7 (7.7%) patients. We observed 12 (33.3%), 7 (14.6%), and 1 (14.3%) deaths in the respective groups.

## 4. Discussion

In the present study we showed that hemodynamic improvement in PAH patients treated with targeted therapy is reflected by favorable changes in several ECG patterns. This referred only to the group without RBBB. Additionally we showed that in this group of patients a change in amplitude of *R* wave in *V*_1_ after targeted treatment is associated with long-term survival.

### 4.1. Electrocardiographic Signs of Pulmonary Hypertension

Structural changes in right atrium and right ventricle such as hypertrophy or dilation as observed in PH are reflected by several ECG patterns [2, 13–20]. Some of them including qR in lead *V*_1_ [21–23], *p* wave amplitude in lead II [21], resting heart rate [24], *p* wave duration [25], precordial electrocardiogram voltage (sum of *R* wave in *V*_1_ and maximum *S* wave amplitude in *V*_5_ or *V*_6_) [26], QRS duration [27], and QTc duration [28] have been shown to have prognostic impact in patients with IPAH, ES, or CTEPH. Despite that, little is known about dynamics of ECG changes during the course of the disease. In a single study of Tonelli et al. [29] electrocardiograms were assessed at time of diagnosis of pulmonary hypertension and before death. The study showed that some ECG parameters altered as the disease progressed. There was an increase in median heart rate; *R* : *S*_*V*1_ ratio; and duration of PR interval, QRS complex, and QTc. Favorable dynamics of electrocardiogram was also shown in our study, but only in the group of patients with significant hemodynamic improvement. On the other hand, patients in whom we did not observe hemodynamic improvement ECG parameters worsened.

### 4.2. Correlations between ECG and Hemodynamics in PAH

Only few studies have evaluated the relationship between surface ECG and hemodynamics in PH. The first study was conducted before the era of PAH specific treatment by Kanemoto in 47 patients with IPAH [8]. In that study the amplitude of *R*_*V*1_ and *R* : *S*_*V*1_ correlated with the pulmonary artery systolic pressure (*r* = 0.46, *p* < 0.01, and *r* = 0.50, *p* < 0.01, resp.), and an amplitude of *R*_*V*1_ > 1.2 mV indicated a pulmonary artery systolic pressure of more than 90 mmHg with a sensitivity of 94% and a specificity of 47%. Additionally, amplitude of the *R*_*V*5_ (*r* = 0.46, *p* < 0.01), *R*_*V*6_ (*r* = 0.46, *p* < 0.01), *R* : *S*_*V*5_ (*r* = 0.39, *p* < 0.01), and *R* : *S*_*V*6_ (*r* = 0.48, *p* < 0.01) correlated with cardiac index. In another study Cheng at al. assessed the relationship between ECG patterns and hemodynamics in 194 IPAH patients [7]. The study showed correlations between *P*_II_ and mPAP (*r* = 0.35, *p* ≤ 0.001) and CI (*r* = −0.22, *p* = 0.002); *R*_*V*1_ and mPAP (*r* = 0.36, *p* ≤ 0.001); *S*_*V*6_ and mPAP (*r* = 0.26, *p* = 0.03) and CI (*r* = −0.22, *p* = 0.003). These studies made the background for the hypothesis that ECG can be a valuable tool to monitor hemodynamics in PH. The correlations between changes in Δ*R*_*V*1_, Δmax⁡*R*_*V*1,2_ + max⁡*S*_I,aVL_ − *S*_*V*1_, and Δ*P*_II_ and hemodynamic parameters were relatively low. Therefore other factors modifying this relationship should be considered, such as diverse dynamics of reversed RV remodeling in response to improving hemodynamics in individual patients.

### 4.3. ECG Changes after Mechanical Reduction of Right Ventricle Overload

ECG changes after reduction of right ventricular overload were well shown in CTEPH patients treated with pulmonary endarterectomy (PEA) and in patients operated for right ventricular outflow tract obstruction (RVOTO). In one cohort of 99 patients with CTEPH who underwent pulmonary endarterectomy (PEA), the decrease of *P*_II_, *R*_*V*1_ and normalization of negative *T*_*V*1–*V*3_ were observed 1 month after PEA. Additional changes such as increase of *S*_*V*1_, increase of *R* : *S*_*V*6_, and decreased prevalence of *S*_I_*Q*_III_ pattern were observed at 1-year follow-up [30]. Of these changes only a decrease in *P*_II_ correlated with lowering of mPAP and PVR. In another study of 30 patients who underwent percutaneous pulmonary valve implantation due to severe RVOTO, the improvement in some ECG patterns such as reduction of *R*_aVR_, *R*_*V*1_, *S*_*V*5_, *S*_*V*6_, and Sokolow–Lyon index correlated with regression in RV mass and volume [9].

### 4.4. Changes of ECG after Targeted Treatment for PAH

So far, only two studies have assessed ECG changes after PAH targeted therapy. In first of them Henkens et al. [31] assessed the relationship between hemodynamic response to treatment and changes of selected ECG parameters such as heart rate, *P* wave amplitude, QRS axis and duration, and *T* wave axis in 81 PAH patients. Hemodynamic response was defined as a decrease of PVR to less than 500 dyne·s·cm^−5^. Within 13.1 months of treatment, the responders had lower values of heart rate and *P* wave amplitude, less rightwards oriented QRS axis, and more rightwards *T* wave axis than the nonresponders. However in another small study of 36 PAH patients, changes in none of the assessed ECG patterns (heart rate, QRS duration, calculated QT interval, premature ventricular contractions, right axis deviation, right bundle branch block, and some measurements of right ventricular hypertrophy) predicted hemodynamic improvement [32]. The aforementioned studies analyzed only a few selected ECG criteria and each of them showed different results. In the present paper we focused on a set of ECG parameters indicating RV overload which were recommended for clinical use in the AHA/ACCF/HRS guidelines [10]. We showed that among them only *R*_*V*1_, *P*_II_, and Δmax⁡*R*_*V*1,2_ + max⁡*S*_I,aVL_ − *S*_*V*1_ are vulnerable to significant changes when hemodynamics of pulmonary circulation improves. We showed that among them only *R*_*V*1_, *P*_II_, and Δmax⁡*R*_*V*1,2_ + max⁡*S*_I,aVL_ − *S*_*V*1_ differentiated patients with and without hemodynamics improvement as presented in Table 3.

In previous studies on the association between ECG changes and hemodynamics in PAH both patients with and without RBBB were enrolled. RBBB, however, changes several patterns in ECG and therefore we supposed that it also could modulate the response of ECG to changing hemodynamics. When analyzing patients with RBBB and without RBBB separately we found that our data refer only to the group without RBBB and consequently that changes in the AHA/ACCF/HRS markers of RV overload are not useful to monitor hemodynamics in PAH patients with RBBB.

In our previous study [2] we showed that *R*_*V*1_ and *P*_II_ were directly related to right ventricular hypertrophy; therefore we think that changes of these parameters during treatment reflect reverse RV remodeling in patients with significant improvement of hemodynamics. The pharmacological effectiveness on reversal of RV hypertrophy in pulmonary hypertension has been shown in animal models [33–35] and in humans treated with pharmacological [36–39] and surgical treatment [30].

## 5. Strengths and Limitations

The main strength of our study is a comprehensive analysis of changes in several ECG patterns of right ventricular hypertrophy and overload in patients with precapillary pulmonary hypertension treated with targeted therapies. We showed a set of parameters (*R*_*V*1_, max⁡*RV*_1,2_ + max⁡*S*_I,aVL_ − *S*_*V*1_, and *P*_II_) which can be used in clinical practice to predict hemodynamic effectiveness of treatment. This can be useful in planning the management of PH patients. We have also shown that the significance of ECG criteria should be interpreted differently in patients with and without RBBB. Additionally we showed that dynamics of ECG in patients with PH can predict prognosis in this group.

Our study has also some limitations. We included patients with PAH of different etiologies and inoperable CTEPH. However, the pathomechanism of RV overload in both conditions is similar. Nevertheless we cannot refer our conclusions to other types of pulmonary hypertension such as PH due to left heart disease or due to pulmonary diseases.

## 6. Conclusions

Electrocardiogram may be useful in predicting hemodynamic effects of targeted therapy in precapillary pulmonary hypertension. Decrease of *R*_*V*1_, max⁡*R*_*V*1,2_ + max⁡*S*_I,aVL_ − *S*_*V*1_, and *P*_II_ correspond with hemodynamic improvement after treatment. Of these changes a decrease of *R* wave amplitude in *V*_1_ is associated with better survival.

## Figures and Tables

**Table 1:** Baseline characteristics of the study group and a comparison of patients regarding hemodynamic improvement.

	Whole group	No hemodynamic improvement	Hemodynamic improvement	*p*
*N*	91	45	46	
Age [years]	52.6 ± 16.4	51.3 ± 16.9	53.8 ± 15.9	0.48
Sex (females)	62 (68.1%)	32 (71.1%)	30 (65.2%)	0.55
Etiology				
IPAH [*n*, (%)]	54 (59.3%)	27 (60%)	27 (58.7%)	0.9
CTD-APAH [*n*, (%)]	16 (17.6%)	8 (17.8%)	8 (17.4%)	0.96
CHD-APAH [*n*, (%)]	10 (11%)	7 (15.6%)	3 (6.5%)	0.17
Inoperable CTEPH [*n*, (%)]	11 (12.1%)	3 (6.7%)	8 (17.4%)	0.12
6MWD [m]	325.5 ± 105.9	328.3 ± 114.8	322.6 ± 97.3	0.8
NT-proBNP [pg/mL]	2608.3 ± 3139.5	2397.5 ± 2358.9	2824.0 ± 3792	0.52
WHO-FC [mean ± SD]	3.08 ± 0.57	3.0 ± 0.58	3.15 ± 0.55	0.2
RHC:				
mRAP [mmHg]	6.8 ± 4.6	6.4 ± 3.8	7.3 ± 5.4	0.39
PAWP [mmHg]	8.1 ± 3.8	8.5 ± 4.0	7.9 ± 3.6	0.47
SpO2 in Ao [%]	91.5 ± 7.3	91.5 ± 7.3	91.6 ± 7.5	0.94
SpO2 in PA [%]	59.7 ± 10.7	59.7 ± 11.7	58.7 ± 9.9	0.99
CI [l/min/m^2^]	1.97 ± 0.59	1.97 ± 0.63	1.97 ± 0.55	0.98
PVR [WU]	14.4 ± 8.3	15.7 ± 9.2	13.2 ± 7.1	0.15
Time between consecutive RHC procedures	12.6 ± 10.0	11.3 ± 8.2	13.9 ± 11.4	0.23

*Abbreviations*. IPAH: idiopathic pulmonary arterial hypertension; CTD-APAH: pulmonary arterial hypertension associated with connective tissue disease; CHD-APAH: pulmonary arterial hypertension associated with congenital heart disease; CTEPH: chronic thromboembolic pulmonary hypertension; 6MWD: 6-minute walking test distance; NT-proBNP: N-terminal pro-B type natriuretic peptide level; WHO-FC: World Health Organization functional class; RHC: right heart catheterization; mRAP: mean right atrial pressure; PAWP: pulmonary artery wedge pressure; SpO_2_: oxygen saturation; Ao: aorta; PA: pulmonary artery; CI: cardiac index; PVR: pulmonary vascular resistance.

**Table 2:** Baseline electrocardiography characteristics.

	No hemodynamic improvement	Hemodynamic improvement	*p*
HR [bpm]	79.0 ± 17.2	76.4 ± 16.4	0.46
Sinus rhythm [*n* (%)]	39 (86.7%)	40 (87.0%)	0.97
*R*_*V*1_ [mm]	6.5 ± 5.7	4.2 ± 3.0	0.02
*R*_*V*1_ > 6 mm [*n* (%)]	17 (37.8%)	13 (28.3%)	0.34
*R* : *S*_*V*1_ [mm]^1^	6.9 ± 12.0	3.2 ± 4.6	0.09
*R* : *S*_*V*1_ > 1.0 [*n* (%)]	36 (80%)	29 (63%)	0.08
*S*_*V*5_ [mm]	9.5 ± 5.5	8.6 ± 4.5	0.39
*S*_*V*5_ > 10 mm [*n* (%)]	17 (37.8%)	17 (37%)	0.94
*S*_*V*6_ [mm]	6.5 ± 4.6	6.1 ± 3.8	0.64
*S*_*V*6_ > 3 mm [*n* (%)]	33 (73.3%)	35 (76%)	0.76
*R*_aVR_ [mm]	3.2 ± 2.3	2.5 ± 2.1	0.17
*R*_aVR_ > 4 mm [*n* (%)]	9 (20%)	5 (10.9%)	0.62
*S*_*V*1_ [mm]	2.6 ± 4.0	2.8 ± 3.4	0.78
*S*_*V*1_ < 2 mm [*n* (%)]	29 (64.4%)	26 (56.5%)	0.44
*R*_*V*5,6_ [mm]	7.9 ± 4.3	7.7 ± 3.3	0.83
*R*_*V*5,6_ < 3 mm [*n* (%)]	3 (6.7%)	3 (6.5%)	0.98
*R* : *S*_*V*5_ [mm]	1.3 ± 1.0	1.8 ± 2.7	0.25
*R* : *S*_*V*5_ < 3 mm [*n* (%)]	15 (33.3%)	12 (26%)	0.45
*R* : *S*_*V*6_ [mm]	1.9 ± 1.9	2.7 ± 4.5	0.31
*R* : *S*_*V*6_ < 3 mm [*n* (%)]	1 (2.2%)	4 (8.7%)	0.18
*R* : *S*_*V*5_ to *R* : *S*_*V*1_ [mm]^1^	3.0 ± 6.4	6.4 ± 19.4	0.35
*R* : *S*_*V*5_ to *R* : *S*_*V*1_ < 0.04 [*n* (%)]^1^	1 (2.2%)	1 (2.2%)	1
(*R*_I_ + *S*_III_) − (*S*_I_ + *R*_III_) [mm]	−8.8 ± 9.5	−5.3 ± 9.9	0.1
(*R*_I_ + *S*_III_) − (*S*_I_ + *R*_III_) < 15 mm [*n* (%)]	44 (97.8%)	45 (97.8%)	0.99
max⁡*R*_*V*1,2_ + max⁡*S*_I,aVL_ − *S*_*V*1_ [mm]	13.5 ± 11.3	9.2 ± 7.2	0.03
max⁡*R*_*V*1,2_ + max⁡*S*_I,aVL_ − *S*_*V*1_ > 6 mm [*n* (%)]	36 (80%)	37 (80.4%)	0.96
*R*_*V*1_ + *S*_*V*5,6_ [mm]	16.3 ± 9.7	12.8 ± 6.1	0.05
*R*_*V*1_ + *S*_*V*5,6_ > 10.5 [*n* (%)]	33 (73.3%)	29 (63%)	0.29
*R*_*V*1_ peak [mm]	40.5 ± 13.8	38.8 ± 20.6	0.54
*R* peak *V*_1_ > 35 msec in baseline^1^ [*n* (%)]	21 (65.6%)	21 (61.2%)	0.75
*P*_II_ [mm]^3^	1.48 ± 0.6	1.7 ± 0.8	0.29
*P*_II_ > 0,25 mV [*n* (%)]^3^	1 (2.6%)	5 (12.2%)	0.11
Qualitative patterns:			
qR in *V*_1_ [*n* (%)]	21 (46.7%)	17 (40%)	0.35
RSR_*V*1_ (QRS duration > 0.12 sec)	3 (6.7%)	4 (8.7%)	0.72
*S* > *R* in I	34 (75.6%)	26 (56.5%)	0.06
*S* > *R* in II	12 (26.7%)	8 (17.4%)	0.29
*S* > *R* in III	7 (15.6%)	9 (15.6%)	0.62
*S*_I_ and *Q*_III_	25 (55.6%)	26 (56.5%)	0.93
*R* : *S*_*V*1_ > *R* : *S*_*V*3,4_	20 (64.5%)	20 (55.6%)	0.46
Negative *T*-wave *V*_1_ through *V*_3_	25 (55.6%)	27 (58.7%)	0.76

^1^Calculated for patients who had all required waves present; ^2^calculated according to guidelines, only for patients with QRS < 120 msec; *n* = 32 and *n* = 34, respectively; ^3^calculated only for patients in sinus rhythm; *n* = 39 and *n* = 40, respectively.

**Table 3:** Changes of quantitative electrocardiographic parameters after PAH specific treatment.

	No hemodynamic improvement	Hemodynamic improvement	*p* ^*∗*^
Δ*R*_*V*1_ [mm]	+1.38 ± 3.9	−0.82 ± 2.0	0.002
Δ*R* : *S*_*V*1_ [mm]	−1.0 ± 4.9^1^	−0.6 ± 1.4^1^	1
Δ*S*_*V*5_ [mm]	+0.17 ± 4.7	−1.1 ± 4.0	1
Δ*S*_*V*6_ [mm]	+1.3 ± 5.6	−0.91 ± 3.9	0.45
Δ*R*_aVR_ [mm]	−0.1 ± 2.0	−0.7 ± 1.9	1
Δ*S*_*V*1_ [mm]	−0.46 ± 3.0	−0.48 ± 2.7	1
Δ*R*_*V*5,6_ [mm]	−0.7 ± 4.4	0 ± 3.1	1
Δ*R* : *S*_*V*5_ [mm]	−0.2 ± 1.2	+0.2 ± 1.2	1
Δ*R* : *S*_*V*6_ [mm]	−0.8 ± 1.9	+0.3 ± 2.8	0.6
Δ*R* : *S*_*V*5_ to *R* : *S*_*V*1_ [mm]	−1.6 ± 4.0^1^	+1.2 ± 4.9^1^	1
Δ(*R*_I_ + *S*_III_) − (*S*_I_ + *R*_III_) [mm]	−2.1 ± 6.9	+0.9 ± 4.1	0.2
Δmax⁡*R*_*V*1,2_ + max⁡*S*_I,aVL_ − *S*_*V*1_ [mm]	+3.4 ± 7.4	−1.0 ± 4.6	0.002
Δ*R*_*V*1_ + *S*_*V*5,6_ [mm]	+1.7 ± 6.7	−1.9 ± 4.3	0.06
Δ*R* peak *V*_1_ [mm]	−2.9 ± 22.2^2^	−11.3 ± 23.4^2^	1
Δ*P*_II_	+0.5 ± 1.0	−0.1 ± 0.7	0.03

^1^Calculated for patients who had all required waves present; ^2^calculated according to guidelines, only for patients with QRS < 120 msec; *n* = 32 and *n* = 34, respectively. ^*∗*^*p* values were mathematically adjusted using Bonferroni correction for multiple comparisons.

**Table 4:** Correlation between changes in ECG criteria and hemodynamic variables.

	ΔPVR		ΔmPAP		ΔCI	
	*R*	*p*	*R*	*p*	*R*	*p*
Δ*R*_*V*1_ [mm]	0.33	0.002	0.21	0.05	−0.23	0.03
Δmax⁡*R*_*V*1,2_ + max⁡*S*_I,aVL_ − *S*_*V*1_ [mm]	0.33	0.002	0.26	0.01	−0.31	0.004
Δ*P*_II_ [mm]	0.23	0.04	0.13	0.26	−0.25	0.03

## Supplementary Materials

Supplementary Materials In supplementary Table S1 we showed how the following ECG parameters changed after PAH specific treatment in patients with and without significant hemodynamic improvement: qR_*V*1_, RSR_*V*1_, *S* > *R* in I, *S* > *R* in II, *S* > *R* in III, *S*_I_ and *Q*_III_, *R* : *S*_*V*1_ > *R* : *S*_*V*3,4_, and negative *T*-wave *V*_1_ through *V*_3_. In supplementary Tables S2–S5 we compared patients with and without hemodynamic improvement with respect to changes in several ECG parameters after PAH specific treatment separately for a subgroup with and without RBBB. Table S1: changes of qualitative electrocardiographic parameters after PAH specific treatment in the whole sample. Table S2: changes of quantitative electrocardiographic parameters after PAH specific treatment in patients without right bundle branch block. Table S3: changes of qualitative electrocardiographic parameters after PAH specific treatment in patients without right bundle branch block. Table S4: changes of quantitative electrocardiographic parameters after PAH specific treatment in patients with right bundle branch block. Table S5: changes of qualitative electrocardiographic parameters after PAH specific treatment in patients with right bundle branch block.
