# Corrigendum to “ECG Markers of Hemodynamic Improvement in Patients with Pulmonary Hypertension”

**DOI:** 10.1155/2018/1541709

**Published:** 2018-08-12

**Authors:** Marcin Waligóra, Anna Tyrka, Piotr Podolec, Grzegorz Kopeć

**Affiliations:** Department of Cardiac and Vascular Diseases, Jagiellonian University Medical College, John Paul II Hospital in Krakow, Pradnicka 80, Kraków, Poland

In the article titled “ECG Markers of Hemodynamic Improvement in Patients with Pulmonary Hypertension” [1], there was an error in the key of Figure 2, which should be corrected as follows: 

## Figures and Tables

**Figure 2:**
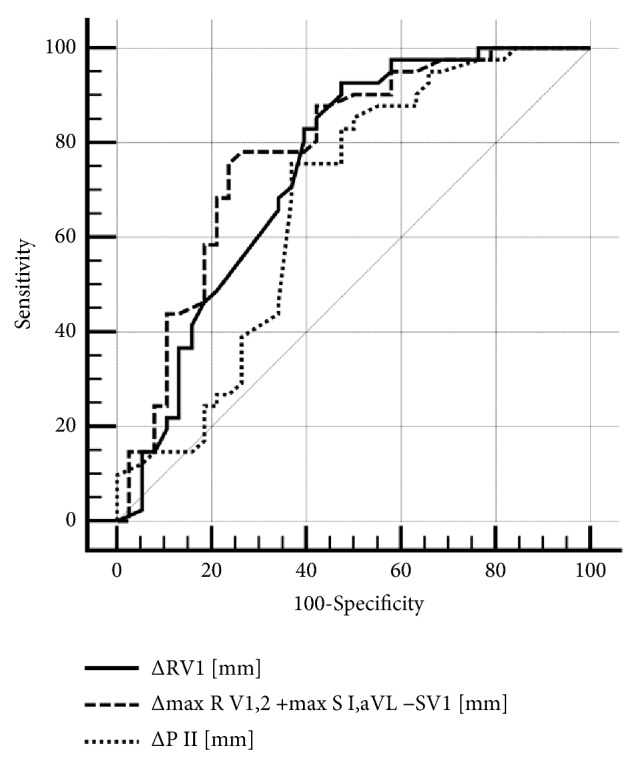
Electrocardiographic predictors of hemodynamic improvement. Δ*R*_*V*1_ (AUC: 0.75, 95% CI: 0.63–0.84, *p* = 0.0005), Δmax⁡*R*_*V*1,2_ + max⁡*S*_I,aVL_ − *S*_*V*1_ (AUC: 0.73, 95% CI: 0.63–0.82, *p* < 0.0001) and Δ*P*_II_ (AUC: 0.67, 95% CI: 0.56–0.77, *p* = 0.007).
