# Supplementary material for: ECG Markers of Hemodynamic Improvement in Patients with Pulmonary Hypertension
Source: Biomed Res Int. 2018 Apr 10;2018:4606053. doi: 10.1155/2018/4606053 (PMC5914124; doi:10.1155/2018/4606053)
Supplement: Supplementary Materials — In supplementary Table S1 we showed how the following ECG parameters changed after PAH specific treatment in patients with and without significant hemodynamic improvement: qRV1, RSRV1, S > R in I, S > R in II, S > R in III, SI and QIII, R : SV1 > R : SV3,4, and negative T-wave V1 through V3. In supplementary Tables S2–S5 we compared patients with and without hemodynamic improvement with respect to changes in several ECG parameters after PAH specific treatment separately for a subgroup with and without RBBB. Table S1: changes of qualitative electrocardiographic parameters after PAH specific treatment in the whole sample. Table S2: changes of quantitative electrocardiographic parameters after PAH specific treatment in patients without right bundle branch block. Table S3: changes of qualitative electrocardiographic parameters after PAH specific treatment in patients without right bundle branch block. Table S4: changes of quantitative electrocardiographic parameters after PAH specific treatment in patients with right bundle branch block. Table S5: changes of qualitative electrocardiographic parameters after PAH specific treatment in patients with right bundle branch block. [file 4606053.f1.docx]

**Supplementary material**

Table S1. Changes of qualitative electrocardiographic parameters after PAH specific treatment in whole sample.

No hemodynamic improvement hemodynamic improvement p^*^

qR in V_1_ :

new [n(%)] 6(13.3%) 1(2.2%)

no change [n(%)] 37 (82.2%) 40 (87%) 0.64

resolution [n(%)] 2(4.4%) 5(10.9%)

RSR_V1_ (QRS duration > 0.12 sec) :

new [n(%)] 0(0%) 0(0%)

no change [n(%)] 45(0%) 44(95.7%) 1

resolution [n(%)] 0(0%) 1(2.2%)

S>R in I:

new [n(%)] 5(11.1%) 5(11.1%)

no change [n(%)] 37(82.2%) 38(82.6%) 1

resolution [n(%)] 3(6.7%) 3(6.5%)

S>R in II:

new [n(%)] 2(4.4%) 0(0%)

no change [n(%)] 39(86.7%) 44(95.7%) 1

resolution [n(%)] 4(8.9%) 2(4.4%)

S>R in III:

new [n(%)] 1(2.2%) 1(2.2%)

no change [n(%)] 39(86.7%) 42(91.3%) 1

resolution [n(%)] 5(11.1%) 3(6.5%)

S_I_ and Q_III_:

new [n(%)] 1(2.2%) 1(2.2%)

no change [n(%)] 41(91.1%) 35(76%) 0.96

resolution [n(%)] 3(6.7%) 10(21.7%)

R:S _V1_ > R:S _V3,4_:

new [n(%)] 1(7.1%)^1^ 1(4.2%)^1^

no change [n(%)] 13(92.9%)^1^ 20(83.3%)^1^ 1

resolution [n(%)] 0(0%)^1^ 3(12.5%)^1^

Negative T-wave V_1_ through V_3_:

new [n(%)] 6(13.3%) 2(4.4%)

no change [n(%)] 37(82.2%) 35(76%) 0.31

resolution [n(%)] 2(4.4%) 9(19.6%)

*p values were mathematically adjusted using Bonferroni correction for multiple comparisons

^1^ calculated only for patient with present waves in according lead both in baseline and follow-up ECG

Table S2. Changes of quantitative electrocardiographic parameters after PAH specific treatment in patients without right bundle branch block.

No hemodynamic improvement hemodynamic improvement p*

N 37 37

ΔR_V1_ [mm] +1.4±3.4 -1.0±1.9 0.008

ΔR:S_V1_ [mm]^1^ -1.1±5.1 -0.7±1.4 1

ΔS_V5_ [mm] +0.55±5.0 -1.3±4.3 1

ΔS_V6_ [mm] +1.1±4.3 -1.1±4.3 0.45

ΔR _aVR_ [mm] +0.1±2.0 -0.9±2.0 0.45

ΔS_V1_ [mm] -0.3±3.3 -0.2±2.5 1

ΔR_V5,6_ [mm] -0.82±4.3 +0.42±3.0 1

ΔR:S_V5_ [mm] -0.3±1.3 +0.4±1.4 0.6

ΔR:S_V6_ [mm] -0.9±1.9 +0.4±3.2 0.6

ΔR:S_V5_ to R:S_V1_ [mm]^1^ -1.4±4.2 +1.7±5.5 0.15

Δ (R_I_ + S_III_) – (S_I_ + R_III_) [mm] -2.7±6.8 +1.1±4.2 0.08

Δmax R V_1,2_ +max S _I,aVL_ –S_V1_ [mm] +3.1±7.3 -1.8±4.0 0.01

ΔR _V1_ + S _V5,6_ [mm] +2.0±6.9 -2.2±4.4 0.03

ΔR peak V_1_ [mm]^2^ -1.9±20.6 -12.1±24.6 0.1

ΔP _II_ +0.3±1.0 -0.26±0.6 0.08

*p values were mathematically adjusted using Bonferroni correction for multiple comparisons

^1^ calculated only for patient with present waves in according lead

^2^ calculated according to guidelines, only for patients with QRS < 120msec

Table S3. Changes of qualitative electrocardiographic parameters after PAH specific treatment in patients without right bundle branch block.

No hemodynamic improvement hemodynamic improvement p^*^

qR in V_1_ :

new [n(%)] 6(16.2%) 1(2.7%)

no change [n(%)] 30(81%) 31(83.8%)

resolution [n(%)] 1(2.7%) 5(13.5%)

RSR_V1_ (QRS duration > 0.12 sec) :

new [n(%)] 0 0

no change [n(%)] 0 0

resolution [n(%)] 0 0

S>R in I:

new [n(%)] 3(8.1%) 5(13.5%)

no change [n(%)] 32(86.5%) 30(81%)

resolution [n(%)] 2(5.4%) 2(5.4%)

S>R in II:

new [n(%)] 2(5.4%) 0

no change [n(%)] 32(86.5%) 35(94.6%)

resolution [n(%)] 3(8.1%) 2(5.4%)

S>R in III:

new [n(%)] 0 1(2.7%)

no change [n(%)] 33(89.2%) 34(91.9%)

resolution [n(%)] 4(10.8%) 2(5.4%)

S_I_ and Q_III_:

new [n(%)] 1(2.7%) 0

no change [n(%)] 34(91.9%) 27(73%)

resolution [n(%)] 2(5.4%) 10(27%)

R:S _V1_ > R:S _V3,4_:

new [n(%)] 1(7.7%) 0

no change [n(%)] 12(92.3%) 16(84.2%)

resolution [n(%)] 0 3(15.8%)

Negative T-wave V_1_ through V_3_:

new [n(%)] 6(16.2%) 2(5.4%)

no change [n(%)] 29(78.4%) 26(70.3%)

resolution [n(%)] 2(5.4%) 9(24.3%)

*p values were mathematically adjusted using Bonferroni correction for multiple comparisons

Table S4. Changes of quantitative electrocardiographic parameters after PAH specific treatment in patients with right bundle branch block

No hemodynamic improvement hemodynamic improvement p*

N 8 9

ΔHR +1.0±11.5 -11.6±17.7 1

ΔR_V1_ [mm] +1.5±2.0 -0.3±6.0 1

ΔR:S_V1_ [mm]^1^ +0.1 -0.4±1.4 1

ΔS_V5_ [mm] -1.6±2.6 -0.1±2.6 1

ΔS_V6_ [mm] +2.2±10.0 -0.4±2.2 1

ΔR _aVR_ [mm] -1.1±1.9 +0.3±1.1 1

ΔS_V1_ [mm] -1.29±1.1 -1.7±3.4 1

ΔR_V5,6_ [mm] -0.2±4.7 -1.7±3.3 1

ΔR:S_V5_ [mm] +0.2±0.6 -0.4±0.6 1

ΔR:S_V6_ [mm] -0.61±2.2 -0.2±0.6 1

ΔR:S_V5_ to R:S_V1_ [mm]^1^ -3.1 -0.5±0.9 1

Δ (R_I_ + S_III_) – (S_I_ + R_III_) [mm] +0.6±7.1 +0.1±3.8 1

Δmax R V_1,2_ +max S _I,aVL_ –S_V1_ [mm] 4.8±8.3 +2.2±5.7 1

ΔR _V1_ + S _V5,6_ [mm] 0±5.4 -0.4±3.3 1

ΔP _II_ +1.2±0.6 0.6±0.6 1

^1^ calculated only for patient with present waves in according lead; n=1 and n=5 respectively

*p values were mathematically adjusted using Bonferroni correction for multiple comparisons

Table S5. Changes of qualitative electrocardiographic parameters after PAH specific treatment in patients with right bundle branch block.

No hemodynamic improvement hemodynamic improvement p^*^

qR in V_1_ :

new [n(%)] 0 0

no change [n(%)] 8(100%) 9(100%)

resolution [n(%)] 0 0

RSR_V1_ (QRS duration > 0.12 sec) :

new [n(%)] 0 0

no change [n(%)] 8(100%) 8(88.9%)

resolution [n(%)] 0 1(11.1%)

S>R in I:

new [n(%)] 2(25%) 0

no change [n(%)] 5(62.5%) 8(88.9%)

resolution [n(%)] 1(12.5%) 1(11.1%)

S>R in II:

new [n(%)] 0 0

no change [n(%)] 7(87.5%) 9(100%)

resolution [n(%)] 1(12.5%) 0

S>R in III:

new [n(%)] 1(12.5%) 0

no change [n(%)] 7(75%) 8(88.9%)

resolution [n(%)] 1(12.5%) 1(11.1%)

S_I_ and Q_III_:

new [n(%)] 0 1(11.1%)

no change [n(%)] 7(87.5%) 8(88.9%)

resolution [n(%)] 1(12.5%) 0

R:S _V1_ > R:S _V3,4_:

new [n(%)] 0 1(20%)

no change [n(%)] 1(100%) 4(80%)

resolution [n(%)] 0 0

Negative T-wave V_1_ through V_3_:

new [n(%)] 0 0

no change [n(%)] 0 0

resolution [n(%)] 0 0

*p values were mathematically adjusted using Bonferroni correction for multiple comparisons
